# Effects of rectal retractor use on dosimetric parameters, treatment-related adverse events, and clinical outcomes of cervical cancer brachytherapy: A retrospective tertiary-centre study

**DOI:** 10.1016/j.ctro.2026.101302

**Published:** 2026-09-30

**Authors:** Ayşe Hazırbulan, Huriye Şenay Kızıltan, Kimia Çepni, Merve Aldıkaçtıoğlu Talmaç, Halime Şule Selman, Ayben Yentek Balkanay, Elif Tekbaş, İlkbal Temel Yüksel

**Affiliations:** a Department of Gynecologic Oncology Surgery, University of Health Sciences, Başakşehir Çam and Sakura City Hospital, Istanbul, Türkiye; b Department of Radiation Oncology, University of Health Sciences, Başakşehir Çam and Sakura City Hospital, Istanbul, Türkiye; c Department of Gynecologic Oncology Surgery, University of Health Sciences, Kanuni Sultan Süleyman Training and Research Hospital, Istanbul, Türkiye; d Private Practice, Obstetrics and Gynaecology, Istanbul, Türkiye; e Department of Medical Physics, Başakşehir Çam and Sakura City Hospital, Istanbul, Türkiye

**Keywords:** Uterine Cervical Neoplasms, Brachytherapy, Radiotherapy Dosage, Organs at Risk, Radiation Injuries, Treatment Outcome, Rectal Retractor

## Abstract

**Background and Purpose:**

We assessed whether rectal retractor use was associated with dose parameters, organ volumes, treatment-related adverse events, and oncological outcomes in patients who underwent cervical cancer brachytherapy.

**Materials and Methods:**

This retrospective study included 148 patients treated with intracavitary brachytherapy between June 2020 and June 2025. Patients treated with and without a rectal retractor were compared regarding dosimetric, volumetric, adverse event, and survival outcomes. Adverse events were graded via RTOG criteria. Cumulative dose parameters were calculated using EQD2. ROC analysis and multivariable logistic regression were performed to evaluate factors associated with grade ≥ 2 adverse events.

**Results:**

Rectal retractor use occurred in 58 patients (39.2%). Baseline demographic characteristics and tumour size were comparable between groups, although rectal retractor use differed according to applicator type. Rectal retractor use was associated with lower rectal D2cc (no retractor vs. retractor: 65.70 Gy vs. 60.79 Gy, *p* = 0.002) but higher bladder D2cc (no retractor vs. retractor: 75.59 Gy vs. 80.08 Gy, *p* < 0.001) values. Prescribed EQD2, HR-CTV coverage, sigmoid D2cc, and volumetric parameters were similar. Grade ≥ 2 vaginal adverse events were less frequent in the retractor group than in the no-retractor group (32.8% vs. 56.7%, *p* = 0.004). No significant difference was observed in grade ≥ 2 rectal (*p* = 0.380) or urinary adverse events (*p* = 0.083). ROC analysis showed that first-fraction volumetric parameters had limited discriminatory ability for grade ≥ 2 adverse events (AUC range: 0.417–0.613). In multivariable analysis, rectal retractor use was associated with lower odds of grade ≥ 2 vaginal adverse events (OR 0.29; 95% CI, 0.13–0.63; *p* = 0.002).

**Conclusions:**

Rectal retractor use was associated with rectal dose sparing and lower odds of grade ≥ 2 vaginal adverse events without compromising target coverage. However, this finding should be interpreted cautiously because rectal retractor placement was not randomised and was influenced by applicator compatibility.

## Highlights

• Rectal retractor use was associated with lower rectal D2cc.
• Rectal retractor use was associated with higher bladder D2cc.
• Rectal retractor use was associated with fewer grade ≥ 2 vaginal adverse events.
• Target coverage and clinical response were similar between groups.
• Retractor use was strongly associated with applicator type; interpret cautiously.

## 1 Introduction

Cervical cancer continues to be a significant global health issue, with mortality rates disproportionately concentrated in low- and middle-income countries. In 2022, approximately 660,000 new cases and 350,000 related deaths were reported worldwide [1], [2]. Regarding locally advanced disease, definitive treatment typically comprises pelvic radiotherapy delivered with concurrent platinum-based chemotherapy, followed by image-guided brachytherapy [3], [4]. Brachytherapy continues to play a central role in curative management, and modern image-guided adaptive planning facilitates the simultaneous assessment of target coverage and organ-at-risk status [3], [5], [6]. In the EMBRACE-I study, in treating locally advanced cervical cancer, the combination of personalised magnetic resonance imaging (MRI)-guided intracavitary and, when necessary, interstitial treatments with three-dimensional treatment planning ensured adequate target volume dose coverage while preserving at-risk organs. This approach in combination with chemoradiotherapy has achieved durable local control in patients with locally advanced cervical cancer. This method has been associated with low rates of serious genitourinary and gastrointestinal adverse events [7], [8]. The EMBRACE-II protocol emphasises that treatment success in modern cervical cancer radiotherapy depends not only on target volume coverage but also on reducing doses to organs at risk (OARs), decreasing treatment duration, and standardising brachytherapy quality [9]. Tandem–ovoid and tandem–ring applicators are among the most commonly employed intracavitary brachytherapy applicators in treating locally advanced cervical cancer [10], [11]. In addition to applicator geometry, rectal retraction methods play a significant role in increasing the radiation source–rectum distance, thereby enhancing treatment plan conformality and reducing the rectal dose [12], [13]. Previous dosimetric studies aiming to compare different applicator geometries and rectal retraction methods, including rectal retractors, vaginal gauze packing, and the tandem Foley balloon, have demonstrated that rectal retractors may yield rectal dose reduction [14], [15]. Although rectal retractors are employed to reduce the rectal dose by increasing the applicator–rectum distance, their effects on volumetric parameters, treatment-related adverse events, and clinical outcomes have not yet been sufficiently characterised. Therefore, this study aimed to evaluate the associations of rectal retractor use with dosimetric parameters, volumetric parameters, treatment-related adverse events, and clinical outcomes of patients with cervical cancer who underwent intracavitary brachytherapy.

## 2 Materials and methods

This retrospective single-centre study included women with cervical cancer who underwent intracavitary brachytherapy between June 2020 and June 2025. Patients treated with definitive radiotherapy, including brachytherapy, were screened for eligibility. Patients with a prior malignancy or prior radiotherapy for the same anatomical region were ineligible. In this retrospective study, eligible patients were classified into rectal retractor and no-retractor groups on the basis of whether a rectal retractor was applied during brachytherapy. Rectal retractor use was determined on the basis of applicator compatibility, patient anatomy, technical feasibility, applicator position, patient tolerance, and treating physician preference. Demographic, clinical, pathological, tumour-related, and treatment-related data of the included patients were retrospectively obtained from the hospital information system and radiation therapy planning records. According to the 2018 International Federation of Gynecology and Obstetrics (FIGO) staging system, stage groups were classified as early, locally advanced, or metastatic. Applicator types were classified as ring, cylinder, or tandem–ovoid. Intracavitary brachytherapy was delivered in multiple fractions according to institutional protocols, most commonly in three fractions. Four- or five-fraction schedules were used in selected patients according to clinical and treatment-planning requirements. Additional fractions were administered to selected patients on the basis of clinical and treatment-planning requirements. All brachytherapy fractions were planned using CT-based image-guided brachytherapy. MRI was used for diagnostic staging and assessment of tumour extent when available, but MRI-guided adaptive planning was not routinely performed for each brachytherapy fraction. In the rectal retractor group, a rectal retractor was used as a standard component of applicator placement and treatment planning for each brachytherapy fraction. Dosimetric evaluation included parameters specific to brachytherapy, expressed as the equivalent dose (EQD2) in 2-Gy fractions, namely, the HR-CTV D90/D98 and D2cc values for the bladder, rectum, and sigmoid colon following cumulative external-beam radiation therapy plus brachytherapy (EBRT+BT). In volumetric analysis, the bladder, HR-CTV, rectum, and sigmoid colon volumes in the first fraction were recorded. Treatment-related adverse events were graded according to Radiation Therapy Oncology Group (RTOG) criteria. For rectal, urinary, and vaginal adverse events, the maximum RTOG grade documented during follow-up for each patient was used for analysis and categorised as grade < 2 versus grade ≥ 2. Because the exact timing of adverse-event onset could not be reliably determined from the retrospective follow-up records, acute and late adverse events were not classified separately. Clinical responses were classified as complete, partial, and absent/progressive responses. Recurrence and survival data were obtained from follow-up records. Survival outcomes included disease-free survival and overall survival (OS).

The study protocol was approved by the Scientific Research Ethics Committee No. 1 of Başakşehir Çam and Sakura City Hospital (decision no.: 223; protocol code: 2025–223; approval date: 13 August 2025). The ethics committee waived the requirement for written informed consent because the analysis was based on anonymised retrospective data.

Statistical analyses were performed in IBM SPSS Statistics version 25.0. Distribution normality was assessed via the Kolmogorov–Smirnov test. Continuous variables are presented as the mean ± standard deviation or median (interquartile range), while categorical variables are presented as counts and percentages. Groups were compared through the Mann–Whitney *U* test, chi-square test, Fisher's exact test, or Fisher–Freeman–Halton exact test, as appropriate. Observed disease-free survival (DFS) and overall survival (OS) values were summarised descriptively using the median and interquartile range. Survival outcomes were assessed via the Kaplan–Meier method and compared using the log-rank test. Multivariable logistic regression was conducted to evaluate factors associated with grade ≥ 2 vaginal adverse events. The model included rectal retractor use, age, stage group, baseline vaginal involvement, and surgical status. Applicator type was not included in the primary model because rectal retractor use was structurally linked to applicator compatibility. Cumulative rectal D2cc was not included in the primary model to avoid adjustment for a potential mediator of the technical intervention. Receiver operating characteristic (ROC) curve analysis was conducted to evaluate the discriminatory performance of first-fraction volumetric parameters for grade ≥ 2 rectal, urinary and vaginal adverse events. Because grade ≥ 2 rectal and urinary adverse events were infrequent, multivariable models for these endpoints were not fitted. A two-sided *p* value <0.05 was considered to indicate statistical significance.

## 3 Results

### 3.1 Patient and tumour characteristics

A total of 148 patients were included. The mean age was 54.3 ± 12.4 years, and the mean body mass index was 27.7 ± 5.9 kg/m^2^. The median longest tumour diameter at the time of brachytherapy was 4.0 cm (IQR, 3.0–5.45). Tumour size at the time of brachytherapy did not differ significantly between the no-retractor and retractor groups [median, 4.3 cm (IQR, 3.0–6.0) vs. 4.0 cm (IQR, 2.7–5.3), respectively; *p* = 0.428]. According to the 2018 International Federation of Gynecology and Obstetrics (FIGO) staging system, 14 patients (9.5%) exhibited early-stage disease, 116 patients (78.4%) exhibited locally advanced disease, and 18 patients (12.2%) exhibited metastatic disease. Stage IIIC1 was the most common FIGO stage. Squamous cell carcinoma was the predominant histology (80.4%). Radiological pelvic lymph node involvement was observed in 61.5% of patients, para-aortic lymph node involvement occurred in 21.6% of patients, and parametrial involvement occurred in 63.5% of patients. Most patients (81.1%) had not undergone surgery. Lymph node dissection was conducted in 14.9% of patients, with pathological nodal positivity in 7 patients. Cylindrical applicators were most commonly adopted (54.1%), and rectal retractor use occurred in 39.2% of patients. Rectal retractor use differed markedly according to applicator type (*p* < 0.001), being used in 84.2% of ring applications and 86.7% of tandem–ovoid applications, but in none of the cylinder applications. Rectal retractor use did not differ significantly according to broad stage group (*p* = 0.098), whereas it was more frequent in patients with baseline vaginal involvement than in those without vaginal involvement (62.1% vs. 33.6%, *p* = 0.005). A palpable pelvic mass was present in 94.6% of patients. In 80.4% of patients, there was no vaginal involvement. When present, upper vaginal involvement was the most common pattern (15.5%). Baseline rectovaginal involvement was present in 7 patients (4.7%) and was suspicious or indeterminate in 1 patient (0.7%) (Table 1).

**Table 1:** Baseline patient, tumour, and treatment characteristics.

**Variable**	**Value**
**Demographic and laboratory characteristics**	
Number of patients	148
Age, years	54.3 ± 12.4
Body mass index, kg/m^2^	27.7 ± 5.9
Gravidity*	3.6 ± 2.5
Parity*	3.0 ± 2.1
Haemoglobin, g/dL	11.5 ± 1.8
C-reactive protein, mg/L†	14.4 ± 22.1
White blood cell count, ×10^9^/L	7.7 ± 3.8
**Tumour characteristics**	
Longest tumour diameter at brachytherapy, cm	4.0 (3.0–5.45)
Clinically documented pelvic mass	140 (94.6%)
**Stage group**	
Early stage	14 (9.5%)
Locally advanced stage	116 (78.4%)
Metastatic stage	18 (12.2%)
**Histopathology**	
Squamous cell carcinoma	119 (80.4%)
Adenocarcinoma	23 (15.5%)
Other histologies	6 (4.1%)
**Radiological pelvic lymph node status**	
Negative	57 (38.5%)
Positive	91 (61.5%)
**Radiological para-aortic lymph node status**	
Negative	116 (78.4%)
Positive	32 (21.6%)
**Baseline parametrial involvement**	
Absent	54 (36.5%)
Present	94 (63.5%)
**Baseline vaginal involvement**	
None	119 (80.4%)
Upper vagina	23 (15.5%)
Lower vagina	3 (2.0%)
Diffuse	3 (2.0%)
**Baseline rectovaginal involvement**	
Absent	140 (94.6%)
Present	7 (4.7%)
Suspicious/indeterminate	1 (0.7%)
**Treatment characteristics**	
**Surgical status**	
No surgery	120 (81.1%)
Any surgical procedure	28 (18.9%)
**Lymph node dissection performed**	
No	126 (85.1%)
Yes	22 (14.9%)
**Variable**	**Value**
**Pathological lymph node status**	
Negative	15 (10.1%)
Positive	7 (4.7%)
Not assessed / no dissection	126 (85.1%)
**Applicator type**	
Cylinder	80 (54.1%)
Ring	38 (25.7%)
Tandem–ovoid	30 (20.3%)
**Chemotherapy status**	
No	14 (9.5%)
Yes	134 (90.5%)
**Chemotherapy regimen‡**	
Cisplatin	102 (77.9%)
Carboplatin-based regimen	21 (16.0%)
Other regimens	8 (6.1%)
**Rectal retractor use**	
No	90 (60.8%)
Yes	58 (39.2%)

Data are presented as mean ± standard deviation or n (%), as appropriate.

* Gravidity and parity data were available for 110 patients.

† C-reactive protein data were available for 142 patients.

‡ Chemotherapy regimen data were available for 131 of 134 patients who received chemotherapy; percentages are calculated among patients with available regimen data.

### 3.2 Clinical response and oncological outcomes

Clinical responses were complete in 107 patients (72.3%), partial in 23 (15.5%), and absent/progressive in 18 (12.2%). During follow-up, recurrence occurred in 52 patients (35.1%), and 41 patients (27.7%) died. The descriptive median observed disease-free survival (DFS) and OS were 20.1 months (IQR, 8.2–35.0) and 26.0 months (IQR, 10.9–38.0), respectively (Table 2).

**Table 2:** Clinical outcomes, follow-up findings, and radiotherapy characteristics (*n* = 148).

**Variable**	**Value**
**Best treatment response**	
Complete response	107 (72.3%)
Partial response	23 (15.5%)
Absent/progressive response	18 (12.2%)
**Oncological events and vital status**	
Disease recurrence (total)	52 (35.1%)
*Local recurrence*	28 (18.9%)
Vital status (Alive at last follow-up)	107 (72.3%)
Vital status (Deceased)	41 (27.7%)
**Posttreatment clinical findings**	
Cervical synechiae or atrophy	96 (64.9%)
Vaginal stenosis present	97 (65.5%)
Vaginal necrosis	6 (4.1%)
Vaginal bleeding	15 (10.1%)
Vesicovaginal fistula	2 (1.4%)
Rectovaginal fistula	5 (3.4%)
Combined fistula	2 (1.4%)
**Follow-up and survival summary (months)**	
Follow-up duration, median (IQR)	26.0 (10.7–37.5)
Observed DFS, median (IQR)	20.1 (8.2–35.0)
Observed OS, median (IQR)	26.0 (10.9–38.0)
**Brachytherapy details**	
*Number of brachytherapy fractions*	
3 fractions	140 (94.6%)
4 fractions	7 (4.7%)
5 fractions	1 (0.7%)
**External-beam radiotherapy details**	
Total number of fractions	25.7 ± 1.8
Dose per fraction, Gy	1.9 ± 0.1
Total dose, Gy	47.7 ± 3.4

Data are presented as mean ± standard deviation, median (interquartile range [IQR]), or n (%), as appropriate. Abbreviations: DFS, disease-free survival; OS, overall survival. Observed DFS and OS values are descriptive summaries and do not represent Kaplan–Meier median survival estimates.

### 3.3 Posttreatment clinical findings

Cervical synechiae/atrophy and vaginal stenosis constituted the most common posttreatment findings. Fistula formation was observed in 9 patients (6.1%). Overall, vaginal posttreatment findings were more frequent than rectal or urinary adverse events. Vesicovaginal fistulas were identified in 1.4% of patients, rectovaginal fistulas occurred in 3.4%, and both vesicovaginal and rectovaginal fistulas occurred in 1.4% of patients (Table 2).

### 3.4 Radiotherapy and dosimetric characteristics

The mean number of external-beam radiotherapy fractions was 25.7 ± 1.8, the dose per fraction was 1.86 ± 0.11 Gy, and the total dose was 47.7 ± 3.4 Gy. EBRT total dose, number of fractions, and dose per fraction did not differ significantly according to rectal retractor use (*p* = 0.085, *p* = 0.250, and *p* = 0.875, respectively). Brachytherapy was delivered in 3 fractions in 140 patients (94.6%), 4 fractions in 7 patients (4.7%), and 5 fractions in 1 patient (0.7%). The brachytherapy fraction number differed according to rectal retractor use (*p* = 0.043), whereas the mean prescribed brachytherapy dose did not differ significantly between groups (*p* = 0.165). The mean cumulative prescribed dose (EBRT+BT) was 77.9 ± 6.1 Gy EQD2 (α/β = 10 Gy). The mean cumulative HR-CTV D98 and D90 values were 75.5 ± 5.6 Gy and 86.0 ± 5.2 Gy, respectively. The mean cumulative D2cc doses were 75.9 ± 9.4 Gy for the bladder, 64.0 ± 9.2 Gy for the rectum, and 61.3 ± 8.9 Gy for the sigmoid colon (EQD2, α/β = 3 Gy). The mean prescribed dose for brachytherapy alone was 29.8 ± 3.7 Gy EQD2 (α/β = 10 Gy). The mean brachytherapy HR-CTV D98 and D90 values were 27.1 ± 5.0 Gy and 37.5 ± 5.4 Gy, respectively. The mean brachytherapy D2cc doses were 28.2 ± 8.8 Gy for the bladder, 16.5 ± 8.1 Gy for the rectum, and 13.7 ± 6.8 Gy for the sigmoid colon (EQD2, α/β = 3 Gy) (Table 3).

**Table 3:** Comparison of dosimetric, volumetric, adverse-event, and clinical response parameters according to rectal retractor use.

**Variable**	**Rectal retractor (−)**	**Rectal retractor (+)**	***p* value**
**Cumulative dosimetric parameters (Gy, EQD2**)			
Total prescribed dose	79.3 (73.6–85.1)	79.3 (71.1–87.5)	0.058
HR-CTV D98	76.0 (71.0–81.0)	76.0 (72.2–79.8)	0.902
HR-CTV D90	86.4 (83.7–89.1)	86.7 (83.6–89.9)	0.384
Bladder D2cc	75.6 (63.8–87.4)	80.1 (70.1–90.1)	**<0.001**
Rectum D2cc	65.7 (53.8–77.6)	60.8 (49.6–72.0)	**0.002**
Sigmoid D2cc	60.6 (49.4–71.8)	63.4 (52.2–74.6)	0.090
**Brachytherapy dosimetric parameters (Gy, EQD2)**			
HR-CTV D98	27.7 (21.3–34.1)	27.6 (22.2–33.0)	0.625
HR-CTV D90	38.4 (32.1–44.7)	37.8 (33.3–42.3)	0.651
Bladder D2cc	27.6 (16.2–39.0)	30.6 (20.4–40.8)	**0.001**
Rectum D2cc	19.4 (7.2–31.6)	12.5 (3.7–21.3)	**<0.001**
Sigmoid D2cc	12.8 (2.9–22.7)	14.8 (6.5–23.1)	0.169
**First-fraction volumetric parameters (cm**^**3**^**)**			
Bladder volume	139.1 (94.6–176.3)	153.3 (98.6–177.7)	0.415
HR-CTV volume	27.3 (20.1–39.2)	33.5 (18.7–42.5)	0.261
Rectum volume	41.8 (26.8–62.0)	47.1 (30.4–69.8)	0.052
Sigmoid volume	31.9 (17.1–51.4)	26.2 (17.2–41.9)	0.588
**Adverse events and clinical response, n (%)**			
Grade ≥ 1 rectal adverse events	22 (24.4%)	6 (10.3%)	**0.033**
Grade ≥ 2 rectal adverse events	12 (13.3%)	5 (8.6%)	0.380
Grade ≥ 1 urinary adverse events	24 (26.7%)	8 (13.8%)	0.063
Grade ≥ 2 urinary adverse events	15 (16.7%)	4 (6.9%)	0.083
Grade ≥ 1 vaginal adverse events	70 (77.8%)	43 (74.1%)	0.611
Grade ≥ 2 vaginal adverse events	51 (56.7%)	19 (32.8%)	**0.004**
Grade ≥ 2 acute haematological adverse events	68 (75.6%)	43 (74.1%)	0.846
Grade ≥ 3 acute haematological adverse events	49 (54.4%)	32 (55.2%)	0.931
Complete response	64 (71.1%)	43 (74.1%)	0.688

Data are presented as median (interquartile range, IQR) or number (percentage). Statistically significant *p* values (<0.05) are indicated in bold.

Abbreviations: EQD2: equivalent dose in 2-Gy fractions; HR-CTV: high-risk clinical target volume; D2cc: minimum dose to the most exposed 2 cm^3^ volume; EBRT: external beam radiotherapy; BT: brachytherapy.

Continuous variables were compared using the Mann–Whitney *U* test, and categorical variables were compared using the chi-square or Fisher's exact test, as appropriate.

### 3.5 Comparative analysis according to rectal retractor use

#### 3.5.1 Baseline comparison

Demographic characteristics and tumour size at the time of brachytherapy were broadly comparable between patients treated with and without a rectal retractor. The median longest tumour diameter at brachytherapy did not differ significantly between the no-retractor and retractor groups [4.3 cm (IQR, 3.0–6.0) vs. 4.0 cm (IQR, 2.7–5.3), respectively; *p* = 0.428]. Broad stage group was also not significantly different according to rectal retractor use (*p* = 0.098). However, rectal retractor use differed markedly according to applicator type (*p* < 0.001), being used in 84.2% of ring applications and 86.7% of tandem–ovoid applications, but in none of the cylinder applications. Rectal retractor use was also more frequent in patients with baseline vaginal involvement than in those without vaginal involvement (62.1% vs. 33.6%, *p* = 0.005). Baseline rectovaginal involvement did not differ significantly according to rectal retractor use (Fisher's exact *p* = 0.426).

#### 3.5.2 Dosimetric and volumetric parameters

Rectal retractor use was associated with significantly lower cumulative rectal D2cc values (no retractor vs. retractor: 65.7 Gy vs. 60.8 Gy, *p* = 0.002) and higher bladder D2cc values (no retractor vs. retractor: 75.6 Gy vs. 80.1 Gy, *p* < 0.001). No significant differences in HR-CTV D90, HR-CTV D98, total prescribed dose, or sigmoid D2cc values were observed (all *p* > 0.05). First-fraction volumetric parameters were similar between groups, suggesting that the observed dosimetric differences were not explained by measured volume differences alone (Table 3).

#### 3.5.3 Brachytherapy-specific dosimetric comparison

A similar trend was observed in the analysis specific to brachytherapy. In patients treated with a rectal retractor, the total brachytherapy rectal D2cc values were significantly lower (12.5 vs. 19.4 Gy, *p* < 0.001), and the total brachytherapy bladder D2cc values were significantly greater (30.6 vs. 27.6 Gy, *p* = 0.001). No significant between-group differences were observed in brachytherapy HR-CTV dose parameters or sigmoid D2cc values (Table 3).

#### 3.5.4 Treatment-related adverse events and clinical response

According to RTOG criteria, rectal and urinary adverse events were rare. No rectal adverse event was recorded in 81.1% of patients, whereas RTOG grade 1, 2, 3, and 4 rectal adverse events occurred in 7.4%, 4.7%, 1.4%, and 5.4% of patients, respectively. Similarly, no urinary adverse event was recorded in 78.4% of patients, whereas RTOG grade 1, 2, 3, and 4 urinary adverse events occurred in 8.8%, 8.1%, 2.7%, and 2.0% of patients, respectively. Vaginal adverse events were more frequent; no vaginal adverse event was recorded in 23.6% of patients, whereas RTOG grade 1, 2, 3, and 4 vaginal adverse events occurred in 29.1%, 33.1%, 10.1%, and 4.1% of patients, respectively. When adverse events were classified as grades <2 and ≥ 2, rectal retractor use was not significantly associated with grade ≥ 2 rectal adverse events (8.6% vs. 13.3%, *p* = 0.380) or grade ≥ 2 urinary adverse events (6.9% vs. 16.7%, *p* = 0.083). In contrast, grade ≥ 2 vaginal adverse events were significantly less frequent in the rectal retractor group than in the no-retractor group (32.8% vs. 56.7%, *p* = 0.004). The complete response rates were similar between the retractor and no-retractor groups (74.1% vs. 71.1%, *p* = 0.688) (Table 3). The distribution of vaginal adverse-event grades differed significantly according to rectal retractor use (Fisher's exact *p* = 0.014), with lower rates of grade ≥ 2 events in the retractor group. A significant difference was also observed in the distribution of rectal adverse event grades (Fisher's exact *p* = 0.024), whereas urinary adverse-event grade distribution did not differ significantly between groups (Fisher's exact *p* = 0.438) (Figs. 1A–C). No significant association was found between rectal retractor use and acute haematological adverse events of grade ≥ 2 or ≥ 3 (all *p* > 0.05) (Table 3). Similarly, the overall distribution of the maximum haematological adverse event grade and laboratory parameters did not differ between groups. No small-bowel adverse events were documented in the available follow-up records.

**Fig. 1:**
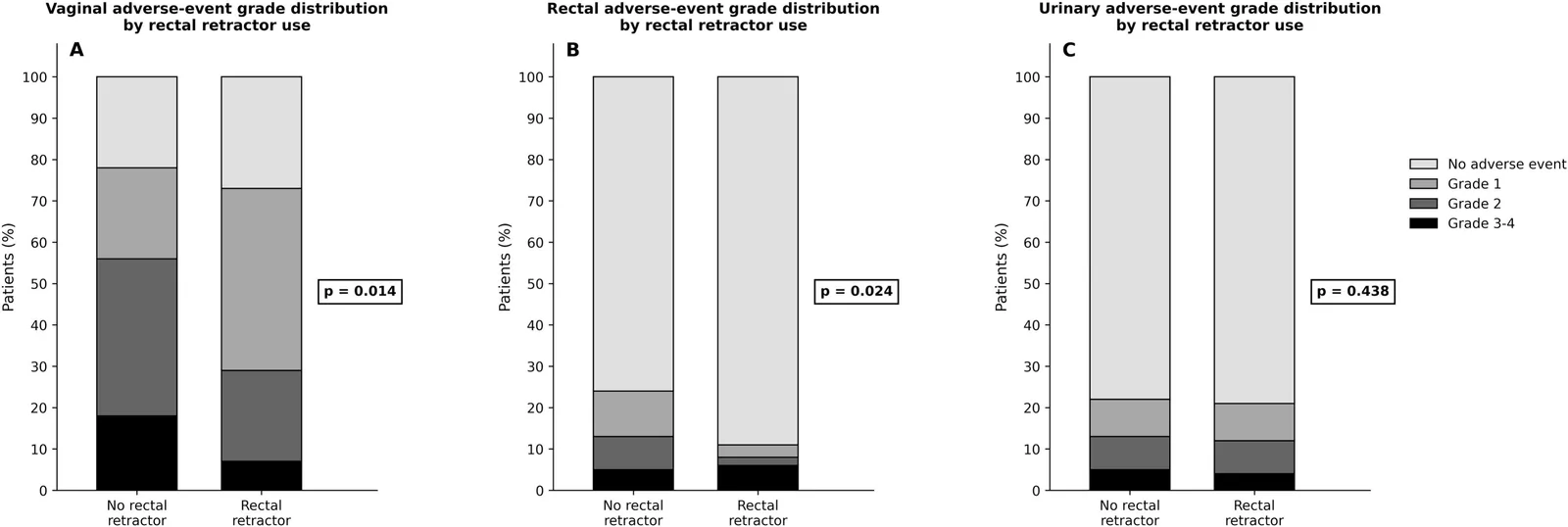
Stacked bar charts showing the distribution of vaginal (A), rectal (B), and urinary (C) adverse-event grades according to rectal retractor use. Grade 3 and 4 adverse events were combined for graphical display. Grade distributions differed significantly for vaginal and rectal adverse events, but not for urinary adverse events (Fisher–Freeman–Halton exact test, *p* = 0.014, *p* = 0.024, and *p* = 0.438, respectively).

#### 3.5.5 Multivariable logistic regression analysis for grade ≥ 2 vaginal adverse events

A multivariable logistic regression model was fitted to evaluate factors associated with grade ≥ 2 vaginal adverse events. The model was statistically significant (omnibus test: χ^2^ = 15.846, degrees of freedom (df) = 6, *p* = 0.015) and showed acceptable calibration (Hosmer–Lemeshow *p* = 0.350). In this exploratory adjusted model, rectal retractor use was associated with lower odds of grade ≥ 2 vaginal adverse events (OR 0.29; 95% CI, 0.13–0.63; *p* = 0.002). Age, stage group, baseline vaginal involvement, and surgical status were not independently associated with grade ≥ 2 vaginal adverse events (Fig. 2).

**Fig. 2:**
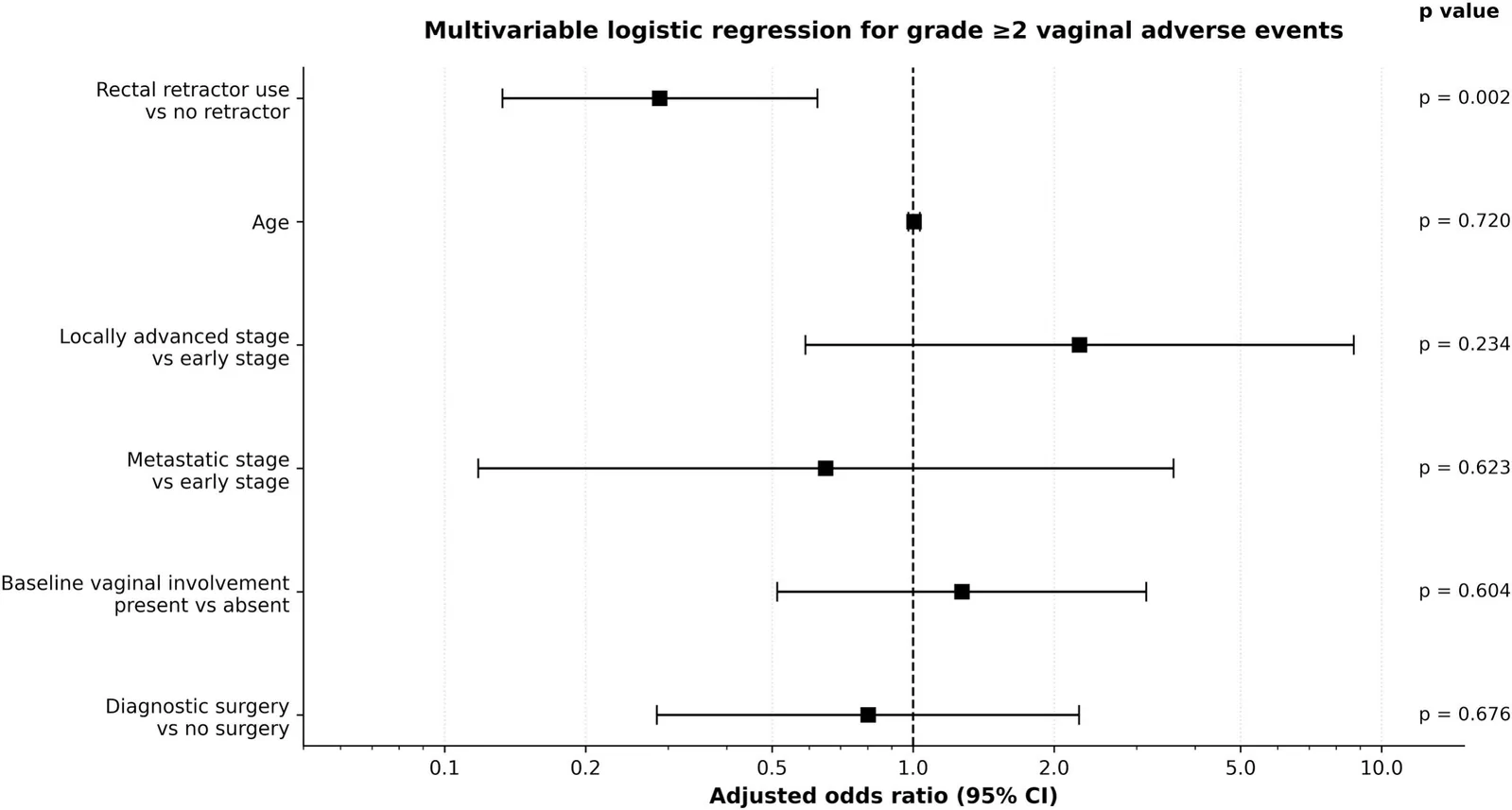
Forest plot of the results of multivariable logistic regression analysis for grade ≥ 2 vaginal adverse events. Adjusted odds ratios (ORs) with 95% confidence intervals (CIs) are shown for rectal retractor use, age, stage group, baseline vaginal involvement, and surgical status. The vertical line indicates an OR of 1.

#### 3.5.6 ROC curve analysis

ROC curve analysis was conducted to evaluate the discriminatory performance of first-fraction volumetric parameters including bladder, HR-CTV, rectal and sigmoid volumes, for grade ≥ 2 adverse events. None of the volumetric parameters showed significant discriminatory ability for grade ≥ 2 rectal adverse events (AUC range: 0.469–0.613), urinary adverse events (AUC range: 0.417–0.562), or vaginal adverse events (AUC range: 0.424–0.564). These findings suggest that first-fraction anatomical volume parameters alone had limited ability to discriminate patients who developed grade ≥ 2 adverse events in this cohort.

#### 3.5.7 Survival analysis

Kaplan–Meier analysis results revealed no significant DFS difference between patients treated with and without a rectal retractor (log-rank *p* = 0.428; Fig. 3A). The estimated mean DFS was 43.34 months (95% CI, 36.54–50.14) in the rectal retractor group and 37.48 months (95% CI, 32.20–42.76) in the no-retractor group. Similarly, OS levels were comparable between groups (log-rank *p* = 0.980; Fig. 3B). The estimated mean OS was 45.34 months (95% CI, 39.02–51.67) in the rectal retractor group and 56.07 months (95% CI, 47.20–64.93) in the no-retractor group.

**Fig. 3:**
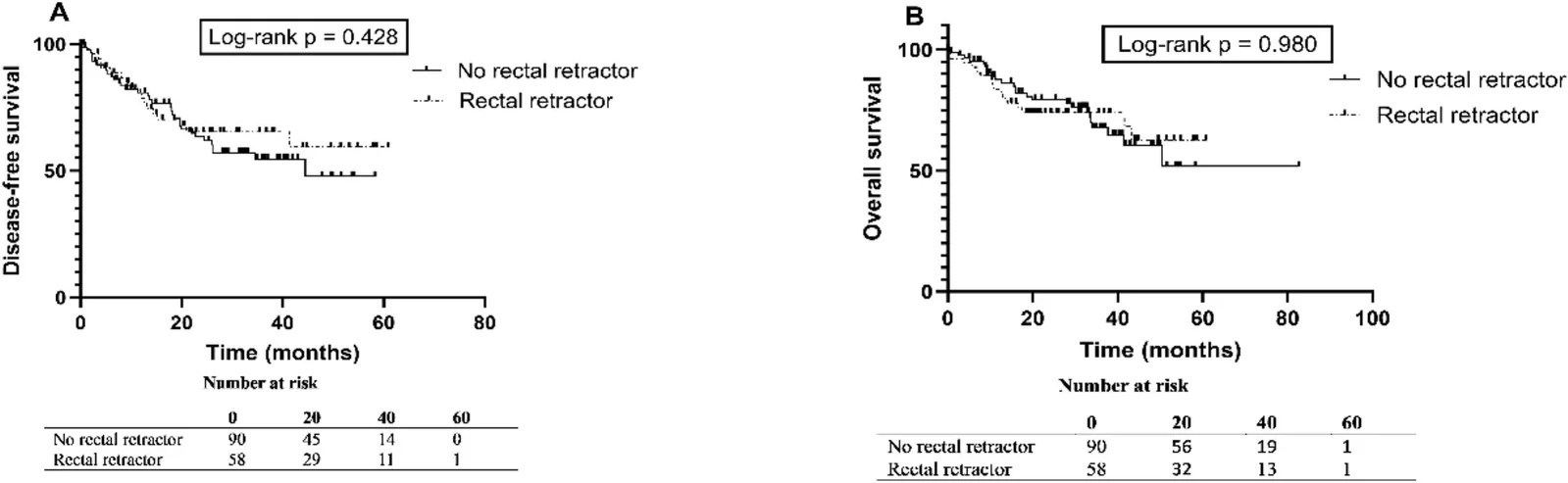
Kaplan–Meier curves according to the use of a rectal retractor. (A) Disease-free survival. (B) Overall survival. No significant differences were observed in disease-free survival (log-rank *p* = 0.428) or overall survival (log-rank *p* = 0.980) between patients treated with and without a rectal retractor.

## 4 Discussion

In this retrospective cohort, we examined whether rectal retractor use was associated with dose distribution, anatomical volume, adverse-event profiles, and oncological outcomes in patients who underwent cervical cancer brachytherapy. The main finding was that rectal retractor use was associated with a lower frequency and lower adjusted odds of grade ≥ 2 vaginal adverse events. However, no significant difference was observed in grade ≥ 2 rectal or urinary adverse events. Image-guided adaptive brachytherapy (IGABT) has become the standard of care for locally advanced cervical cancer, allowing improved target coverage and organ-at-risk (OAR) sparing [13], [16]. Optimisation of dose regimens has reduced severe rectal and urinary adverse events; however, vaginal morbidity remains clinically relevant because of its potential impact on long-term quality of life [8], [17], [18]. Recent EMBRACE-I data also highlight that brachytherapy applicator and implant type should be considered when interpreting morbidity and local-control outcomes, supporting the need to account for applicator-related differences in studies evaluating technical components of brachytherapy [8]. In our study, vaginal adverse events were more frequent than rectal or urinary adverse events. Rectal retractors have primarily been investigated as a method for reducing rectal dose by physically increasing the distance between the rectum and the radiation source [14], [19]. Patient-related factors may also influence the rectal dose distribution [20], [21]. Previous studies have revealed that rectal retraction techniques may alter rectal and bladder dose parameters [12], [19], [22]. Consistent with previous findings, rectal retractor use in our study was associated with lower rectal D2cc values, while HR-CTV coverage and sigmoid D2cc values remained similar between groups. Grade ≥ 2 rectal adverse events were infrequent and did not differ significantly according to rectal retractor use. This situation may reflect the risk of rectal adverse events, which is maintained at a low level because of current dose limitations. Kazoba et al. emphasised that applicator position differences between fractions and the anatomical characteristics of OARs may influence the doses delivered to the rectum, bladder, and sigmoid colon during fractionated intracavitary brachytherapy [13]. Therefore, the clinically measurable contribution of additional dose-reduction techniques may be limited.

In contrast, grade ≥ 2 vaginal adverse events were observed less frequently in patients treated with a rectal retractor. Although the underlying mechanism could not be directly revealed in this study, it may be hypothesised that the rectal retractor increases the distance between the posterior vaginal wall and high-dose regions by altering the applicator geometry. This change may reduce exposure of vaginal tissues to high-dose regions, particularly in the posterior compartment. Sud et al. and Gaudet et al. demonstrated that vaginal tamponade and rectal retraction can modify the position of pelvic organs and, consequently, affect dose distributions during intracavitary brachytherapy [23], [24]. The International Commission on Radiation Units and Measurements (ICRU) recto-vaginal reference point, derived from the conventional ICRU rectal point, has also been associated with grade ≥ 2 vaginal stenosis, supporting a dose–effect relationship between vaginal dose exposure and subsequent vaginal morbidity [25]. This finding provides additional context for the observed association between rectal retractor use and vaginal adverse events in our study, although direct vaginal dose–volume parameters were not available. The limited discriminatory performance of first-fraction volumetric parameters suggests that measured anatomical volumes alone do not explain the observed association with vaginal adverse events. In the exploratory multivariable model, rectal retractor use remained associated with lower odds of grade ≥ 2 vaginal adverse events after adjustment for age, stage group, baseline vaginal involvement, and surgical status. Furthermore, previous studies, including that of Ma et al., have demonstrated that the dosimetric distributions of tandem ring and tandem–ovoid applicators may differ. Therefore, applicator geometry should be considered when interpreting adverse-event outcomes [26]. In the multivariable model, stage group was not independently associated with grade ≥ 2 vaginal adverse events, although disease extent remains clinically relevant when interpreting vaginal morbidity. Although rectal retractor use remained associated with lower odds of grade ≥ 2 vaginal adverse events in the adjusted model, this finding should not be interpreted causally because rectal retractor placement was not randomised and was strongly influenced by applicator compatibility. Junhong et al. and Balsdon et al. demonstrated that applicator stability and treatment-related positional changes during brachytherapy for cervical cancer may affect the reproducibility of dose-related parameters [27], [28].

Importantly, no small-bowel adverse events were documented in the available follow-up records, and all cumulative dose parameters remained within accepted tolerance limits. Furthermore, clinical response rates were similar between patients treated with and without a rectal retractor, suggesting that rectal retractor use was not associated with compromised short-term clinical response. Ohno et al. and Otani et al. reported similar findings from CT-based IGABT studies and demonstrated that acceptable dose-volume parameters can be maintained without compromising treatment effectiveness [29], [30].

This study exhibits several limitations. First, because rectal retractor use was not mandatory per the protocol and depended on physician preference, applicator suitability, patient anatomy, and technical feasibility, even though the baseline characteristics were generally comparable and multivariable adjustments were applied, selection bias and residual confounding cannot be eliminated. This limitation is particularly relevant because rectal retractor use was strongly influenced by applicator type. Second, adverse event assessment was conducted retrospectively and reported by physicians using RTOG criteria. The exact timing of rectal, urinary, and vaginal adverse-event onset could not be reliably determined from the retrospective follow-up records, precluding separate evaluation of acute and late toxicity. Therefore, compared with the actual value, vaginal morbidity may have been under- or overestimated. In addition, given the retrospective design, incomplete ascertainment of small-bowel adverse events cannot be excluded. Third, direct vaginal dose-volume assessment and vaginal wall dose mapping were not conducted, which limits the interpretation of the observed association in terms of mechanism. Finally, the small number of grade ≥ 2 rectal and urinary adverse events limited the feasibility of endpoint-specific multivariable modelling, and uncertainties in the target/OAR definition may have affected dose–volume estimates, particularly in computed tomography (CT)-guided brachytherapy [31], [32]. Because MRI-guided adaptive planning was not routinely performed for each brachytherapy fraction, soft-tissue definition may have been less precise than in fully MRI-guided workflows. Prospective studies incorporating vaginal dose assessment and patient-reported outcomes are needed.

Finally, given the retrospective study design, heterogeneity in disease stages, and relatively short follow-up period, the survival analysis results should be interpreted with caution. Therefore, the absence of a survival difference between groups should not be interpreted as evidence of oncological equivalence.

In conclusion, rectal retractor use was associated with rectal dose sparing, higher bladder D2cc values, and lower odds of grade ≥ 2 vaginal adverse events, without an observed compromise in target coverage or clinical response. However, this association should be interpreted cautiously because rectal retractor placement was not randomised and was influenced by applicator compatibility. These findings suggest that rectal retractors may have a supportive role in optimising the dosimetric profile of intracavitary brachytherapy in selected patients with cervical cancer.

## CRediT authorship contribution statement

**Ayşe Hazırbulan:** Writing – review & editing, Writing – original draft, Visualization, Validation, Supervision, Project administration, Methodology, Investigation, Formal analysis, Data curation, Conceptualization. **Huriye Şenay Kızıltan:** Writing – original draft, Validation, Supervision, Resources, Project administration, Methodology, Investigation, Conceptualization. **Kimia Çepni:** Writing – original draft, Validation, Supervision, Resources, Investigation. **Merve Aldıkaçtıoğlu Talmaç:** Writing – original draft, Validation, Resources, Investigation. **Halime Şule Selman:** Writing – original draft, Validation, Resources, Investigation. **Ayben Yentek Balkanay:** Writing – original draft, Validation, Resources, Investigation. **Elif Tekbaş:** Writing – original draft, Visualization, Validation, Methodology, Formal analysis, Data curation. **İlkbal Temel Yüksel:** Writing – original draft, Validation, Resources, Investigation.

## Ethics approval and consent to participate

This retrospective study was approved by Başakşehir Çam and Sakura City Hospital Scientific Research Ethics Committee No. 1 (approval no: 2025–223; approval date: 13 August 2025). The study was conducted in accordance with the Declaration of Helsinki, and the ethics committee waived the requirement for informed consent owing to the retrospective design.

## Ethics declaration

### Informed consent and patient details

The authors have NOT obtained informed consent from participants or their legal representatives. The requirement for informed consent was waived by the ethics committee because this was a retrospective study based on anonymised patient data.

### Studies in Human

This study was performed in compliance with relevant laws, regulatory frameworks and guidelines where the research took place. This study was approved by the Başakşehir Çam and Sakura City Hospital Scientific Research Ethics Committee No. 1. (Approval No. 2025–223).

## Funding

This research received no specific grant from any funding agency in the public, commercial, or not-for-profit sectors.

## Declaration of competing interest

The authors have no conflicts of interest to disclose.

## Data Availability

The data underlying the results of this study are available from the corresponding author.
