# Supplementary material for: Effects of rectal retractor use on dosimetric parameters, treatment-related adverse events, and clinical outcomes of cervical cancer brachytherapy: A retrospective tertiary-centre study
Source: Clin Transl Radiat Oncol. 2026 Sep 30;62:101302. doi: 10.1016/j.ctro.2026.101302 (PMC13658121; doi:10.1016/j.ctro.2026.101302)
Supplement: Supplementary file 1 [file mmc1.pdf]

Editing Certificate

This document certifies that the manuscript

Effects of rectal retractor use on dosimetric parameters, toxicity, and clinical outcomes of cervical cancer brachytherapy: A retrospective tertiary-centre study

prepared by the authors

Ayşe Hazırbulan, on behalf of all authors

was edited for proper English language, grammar, punctuation, spelling, and overall style by one or more of the highly qualified English speaking editors at AJE.

This certificate was issued on **May 6, 2026** and may be verified on the AJE website using the verification code **6458-0FA4-6A04-6E12-938P** .

Neither the research content nor the authors' intentions were altered in any way during the editing process. Documents receiving this certification should be English-ready for publication; however, the author has the ability to accept or reject our suggestions and changes. To verify the final AJE edited version, please visit our verification page at aje.com/certificate. If you have any questions or concerns about this edited document, please contact AJE at support@aje.com.

AJE provides a range of editing, translation, and manuscript services for researchers and publishers around the world.
For more information about our company, services, and partner discounts, please visit aje.com.
