# Supplementary material for: Effects of rectal retractor use on dosimetric parameters, treatment-related adverse events, and clinical outcomes of cervical cancer brachytherapy: A retrospective tertiary-centre study
Source: Clin Transl Radiat Oncol. 2026 Sep 30;62:101302. doi: 10.1016/j.ctro.2026.101302 (PMC13658121; doi:10.1016/j.ctro.2026.101302)
Supplement: Supplementary file 2 [file mmc2.docx]

**Supplementary Table 1.** Detailed FIGO stage distribution.

| **FIGO stage** | **n (%)** |
| --- | --- |
| IA1 | 1 (0.7%) |
| IB1 | 2 (1.4%) |
| IB2 | 6 (4.1%) |
| IB3 | 5 (3.4%) |
| IIA | 5 (3.4%) |
| IIB | 37 (25.0%) |
| IIIB | 2 (1.4%) |
| IIIC1 | 47 (31.8%) |
| IIIC2 | 19 (12.8%) |
| IVA | 6 (4.1%) |
| IVB | 18 (12.2%) |

Data are presented as n (%). FIGO, International Federation of Gynecology and Obstetrics.
